# Supplementary material for: A Stellar magnesium to silicon ratio in the atmosphere of an exoplanet
Source: Nat Commun. 2026 Feb 18;17:2902. doi: 10.1038/s41467-026-69610-x (PMC13031952; doi:10.1038/s41467-026-69610-x)
Supplement: Supplementary file 1 — SI [file 41467_2026_69610_MOESM1_ESM.pdf]

Supplementary Information for: "A Stellar Magnesium to Silicon ratio in the atmosphere of an exoplanet"

Jorge A. Sanchez*[1], Peter C. B. Smith[1], Krishna Kanumalla[1], Luis Welbanks[1], Michael R. Line†[†1], Stefan Pelletier[2], Steven Desch[1], Patrick Young[1], Jennifer Patience[1], Jacob Bean[3], Matteo Brogi[4,5], Dan Jaffe[6], Gregory N. Mace[6], Megan Weiner Mansfield[7], Vatsal Panwar[8,9], Vivien Parmentier[10], Lorenzo Pino[11], Arjun Baliga Savel[7], Lennart van Sluijs[12], Joost P. Wardenier[13]

[1]*School of Earth and Space Exploration, Arizona State University, Tempe, AZ, USA*
[2]*Observatoire astronomique de l'Université de Genève, Switzerland*
[3]*Department of Astronomy and Astrophysics, University of Chicago, Chicago, IL, USA*
[4]*Dipartimento di Fisica, Università degli Studi di Torino, Torino, Italy*
[5]*INAF – Osservatorio Astrofisico di Torino, 10025 Pino Torinese, Italy*
[6]*Department of Astronomy, The University of Texas at Austin, Austin, TX, USA*
[7]*Department of Astronomy, University of Maryland, College Park, MD, USA*
[8]*Department of Physics, University of Warwick, Coventry, UK*
[9]*Center for Exoplanets and Habitability, University of Warwick, Coventry, UK*
[10]*Laboratoire Lagrange, Observatoire de la Côte d'Azur, CNRS, Université Côte d'Azur, Nice, France*
[11]*INAF – Osservatorio Astrofisico di Arcetri, Florence, Italy*
[12]*Department of Astronomy, University of Michigan, Ann Arbor, MI, USA*
[13]*Trottier Institute for Research on Exoplanets (iREx), Université de Montréal, Montréal, QC, Canada*

Supplementary Note 1: Sources of Opacity Line Lists

The opacity line-lists used in the main analysis come from the following sources; CIA from [1]; H_2O from EXOMOL [2]; CO and OH from the HITEMP database [3, 4]; neutral atomic species from the Kurucz line database (gfall2017) [5], H^- from [6].

Supplementary Discussion

This work joins a growing body of literature studying WASP-189b at high spectral resolution. One of the most complete chemical inventories of WASP-189b was measured by [7] based on optical transmission data from HARPS and HARPS-N [8]. In contrast to our work, Ref. [7] use a free chemistry modeling

*Corresponding author: jasanchez@asu.edu
†Corresponding author; senior author / supervised the project: mrline@asu.edu

prescription in which the volume mixing ratio of each individual gas species is assumed to be constant with pressure. Due to the observed geometry of their analysis (transmission spectrum compared to direct thermal emission), the contribution of their signal originates from the upper atmosphere of the planet, typically probing pressures of 0.1 mbar above an opacity deck [7]. As such, comparison to their work is nontrivial. However, by placing the retrieved enrichment of Fe and Mg from our analysis back into `fastchem`, the code outputs the inferred volume mixing ratios of each gas as a function of pressure. While our analysis is most sensitive to pressures around 0.1 bar, and the transmission spectra of [7] had an opacity deck of 0.1 mbar, the Fe and Mg volume mixing ratios from our main analysis are consistent within the uncertainties to the constant with altitude volume mixing ratios measured by [7].

Ref. [9] observed WASP-189b during eclipse phases using the CRIRES+ instrument. In their analysis, they perform a chemical equilibrium retrieval similar to ours, (albeit without retrieving for the individual gas enrichment), and find the atmosphere of WASP-189b to be metal enriched, at $[\mathrm{M/H}]_\odot = 1.40\ ^{+1.39}_{-0.60}$, while finding a C/O ratio of $0.32\ ^{+0.41}_{-0.14}$. This measurement in metallicity is disparate from our own measurement at 3σ confidence, and we attribute this distinction to differences in each analysis, including the wavelength coverage of the data and how the chemical composition of the atmosphere is parameterized. In particular, Ref. [9] parametrizes the atmosphere with [M/H] (total metallicity defined above) parameter and the Carbon-to-Oxygen ratio. Ref. [9] discusses that the retrieved Oxygen enrichment is linked to the Iron enrichment ([O/H] = [Fe/H]) in this parameterization, meaning that there exists a degeneracy between these parameters as a higher metallicity (favored by their model) is correlated with a lower C/O since these values result in low CO abundance. The non-detections of other Oxygen bearing species such as OH or H_2O in their K-band observations would suggest that their metallicity constraint is primarily driven by the Iron abundance. In contrast, our abundance parameterization, which includes single elemental enrichment factors (e.g., (Fe, Mg, Si...C, O)/H vs. just M/H and C/O) , favors an enrichment of Iron relative to Carbon and Oxygen, which in context of [9] could lead to a high metallicity and low C/O. Given these differences, it is non-trivial to draw further conclusions from the discrepancies in the two results.

The posterior probability distribution from our main result is summarized in Supplementary Figures 3 - 5. A full corner plot showing all 22 parameters is also available in the zenodo repository given in the Data Availability section. Supplementary Figure 6 shows the median P-T profile along with 1 and 2 σ confidence interval generated from 1000 random draws of the posterior probability distribution. We also compare our retrieved profile with another model generated under 1D radiative-convective-thermochemical equilibrium, similar to ref's [10, 11, 12], and find that they are in strong agreement with another.

All the gases in our chemical parametrization show bounded measurements with their 68% confidence intervals. While this is expected given our strong detections in the CCF maps for most species, we note that the constraints achieved on Ti I, Ca I, V I can be also be attributed to their stronger detections using the likelihood function, as seen in the logL S/N maps on bottom row Supplementary Figure 2. As noted in previous HRCCS studies such as refs [13] and [14], species with weak-to-non detections using traditional CCF maps have had constrained abundance measurements within a retrieval framework

due to the greater sensitivity of the log-likelihood function to line amplitudes and line shapes. Because the gases are output from the retrieval based on equation 1 of the main text (relative to the solar values from ref [15]) we calculate the $\log10(\frac{X}{H})$ value for each individual gas by adding the $\log10(\frac{X}{H})$ solar value from [15]. This allows us to calculate relative quantities such as the carbon-to-oxygen and refractory-to-refractory ratios, but to also to compare these derived quantities in the planet with literature stellar values.

As mentioned in the main text, our main source of stellar chemical abundances is cited from ref [16]. For all species with listed stellar measurement uncertainties, we propagate these uncertainties into our calculations by generating arrays of random values from a normal distribution centered at the stellar abundance, with a standard deviation the size of the measurement uncertainty from [16]. We use these arrays as the stellar values from which to compare to our planet measurements. Since the listed C and O abundance in WASP-189 from [16] are presented without uncertainties, we apply an uncertainty of 0.13 dex for each, the same uncertainty level listed for the [Fe/H] value.

We note the difficulty in measuring stellar abundances, and in particular for A type stars such as WASP-189. There exist other measured stellar chemical patterns for the host star besides those presented in [16], such as from [17] and [18], the latter whose work was done in an effort to try and mitigate the rotational effects on the stellar spectrum of WASP-189. However, not all of retrieved gases in our model have measured stellar values in these studies. We therefore elect to quote the values listed [16] for its measurement of both refractory elements as well as Carbon and Oxygen. Should the stellar parameters be updated in the future, the updated calculations of planet-to-stellar ratios is straightforward.

The first column of 1 shows each gas considered in our model, followed by the log abundance of this element relative to Hydrogen in the atmosphere of WASP-189b as measured in our retrieval analysis. Subsequent columns include the $\log_{10}$(X/H) ratio for each element X with respect to solar or stellar literature values for the abundances of these species. Column 3 contains the retrieved enrichment relative to the solar value [15] $[X/H]_{\odot}$ (main output of the retrieval). Column 4, $[X/H]_{*L20}$, are these values relative to the abundances presented in [16], which is the stellar abundance pattern we consider in our main results. Column 5 shows the elemental enrichment relative to [17], $[X/H]_{*S21}$, and finally column 6 from ref [18], $[X/H]_{*La21}$. When the stellar abundance is listed with an uncertainty, we propagate the error bar into our $[X/H]$ value as done with the values from [16] in our main result. If the species is not reported in the respective reference, it is not listed in the table.

As a robustness check for our measured Mg/Si ratio on WASP-189b, we derive the $\log_{10}\left(\frac{(\mathrm{Mg/Si})}{(\mathrm{Mg/Si})_{\star}}\right)$ against some of the different Mg/Si measurements referenced in the main text. These include the solar value [19], the stellar values reported from both [16, 17] and the local FGK population. This is shown in Supplementary Figure 7. Our calculated Mg/Si ratio is consistent against different sets of measured stellar abundances containing both Mg and Si, as well as the solar and local stellar deviations.

The pressure-temperature profile scheme adopted in our analysis has been utilized previously in [14], albeit with a slight different configuration. As predicted from the day-side of ultra-hot Jupiters [20], our retrieval favors a ther-

mal inversion, where the temperature begins to increase with pressure past a certain point. This is consistent with secondary eclipse measurements of ultra-hot Jupiters [21, 14, 22] including analysis previous performed on WASP-189b [23, 9].

Our thermal structure favors a highly irradiated day-side where we would not expect cloud or condensate formation to effect our abundance constraints. To illustrate this point, in Supplementary Figure 8 we plot our pressure-temperature profile from Supplementary Figure 6 alongside condensation curves for major condensates containing Fe, Si,Mg ,Ti and Ca calculated using our retrieved metallicity and the P-T relations from ref's [24, 25]. Our profile does not cross any of these major condensate lines, suggesting that our abundances constraints are not biased by Fe, Mg or Si condensing in the day of the planet. As a further check, we also plot day-side and night-side PT profiles as output from the Global Circulation Models (GCM) for WASP-189b computed in [26]. These models account for 3 dimensional effects on exoplanet atmospheres to understand the chemistry and climate processes on these planets. Encouragingly, our retrieved thermal profile is in agreement with the day-side TP profile from the post-proceeded WASP-189b GCM. Moreover, the night-side TP profile is predicted to be hot enough to also not cross these condensation curves of Fe,Mg or Si bearing species meaning that even the night-side of the planet should not be effected by any major cold-trapping of Fe, Si or Mg. This is further supported by the detection and measurement of these neutral Fe and Mg in transmission [7, 8, 27].

We determine the volatile-to-refractory ratio (R/V) by adding the contributions from volatile (O and C) and refractory (Fe, Si ,Mg ,Ti , Ca , V) elements. Upon doing so, we obtain a slightly super-stellar refractory to volatile ratio of $[\mathrm{R/V}]_* = 0.38^{+0.18}_{-0.19}$, with individual refractory to oxygen and refractory to carbon ratios also slightly super-stellar. Fig. 9 shows how our measured refractory and volatile content coincide with the disk modeling framework developed by [28] to predict the formation location of giant planets. This shows the total sum of the refractory species relative to Hydrogen (relative to stellar) against oxygen (panel a, left) and carbon (panel b, right) to total refractory content (again relative to stellar). The different colored lines correspond to predictions from [28] of these values based on formation location as designated by the legend on the top. Our 2D histograms of these quantities derived from our retrieval results are plotted alongside these colored lines.

Given the measured $[\mathrm{R/H}]_*$ of $0.01^{+0.36}_{-0.29}$, which is a proxy for the solid-to-gas accretion, along with our metallicity and C/O ratios being consistent with the stellar values 68% confidence, our results are consistent within a number of formation pathways within the disk modeling framework presented in ref [28]. The stellar $[\mathrm{R/H}]_*$ value is indicative of a balanced mix of solid and gas accretion, while the moderately O/R and C/R ratios suggest that these solids were slightly elevated in rocky solids as compared to ices. These values are consistent with formation interior to the snow lines, where the planetesimal pollution would be more rich in refractory species than volatiles.

Formation interior to the CO snow line is also supported by our stellar/solar value for the ratio of Carbon-to-Oxygen. However, given that our measured R/V ratio is consistent with the stellar value at 2σ, we cannot rule out formation beyond the CO snow line. The latter case would require WASP-189b to migrate 10's of AU after formation to its final orbital location of 0.05053 ± 0.00098 AU.

Such a large migration pathway toward the interior of the disk may have given rise to the near-polar orbit (the obliquity 86.4 $^{+2.9}_{-4.4}$ degrees, [16]) seen in the WASP-189b system, although there are several mechanisms that are thought to cause the misaligned spin-orbit angles seen many giant planets [29, 30, 31, 32].

Ultimately, determining the exact location of the formation for hot Jupiters remain a non-trivial task, as migration through different disk regions and the evolution of the disk through time are factors that would also need to be considered. More modeling would be required to consider these factors when attempting to reconstruct the formation history of such a unique system, and underscores the difficulty of using present-day gas abundance measurements to estimate the formation pathways of giant planets. To more rigorously address planet formation trends, such measurements over a larger sample of planets is needed.

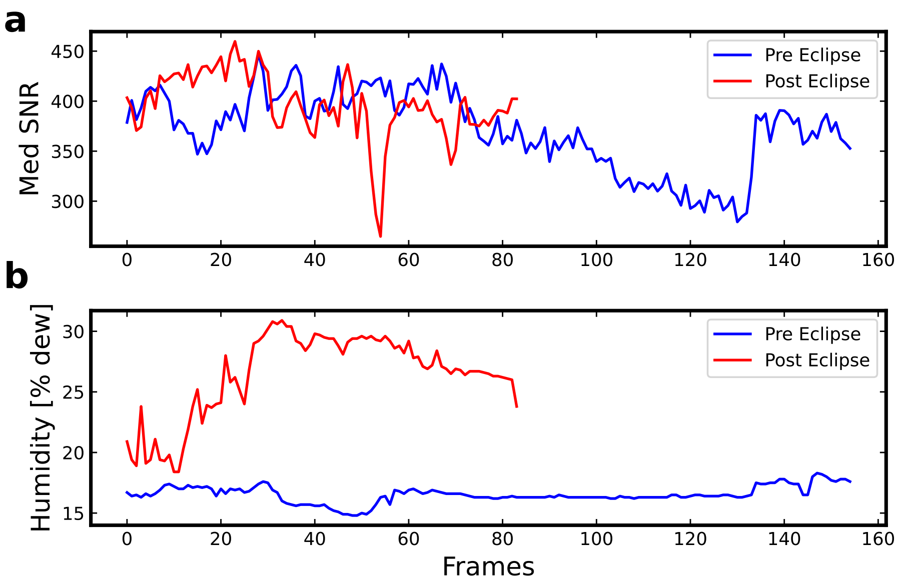

Supplementary Figure 1: **Observing conditions during each night. a** Median observed SNR during each frame of the pre (blue) and post (red) eclipse sequences. **b** Humidity during the observations for the pre (blue) and post (red) eclipse observations. Each frame refers to an AB pair in the AB-BA nodding pattern during observations. In total there were 155 frames for the pre eclipse sequence and 84 for the post eclipse sequence. Source data are provided as a Source Data file.

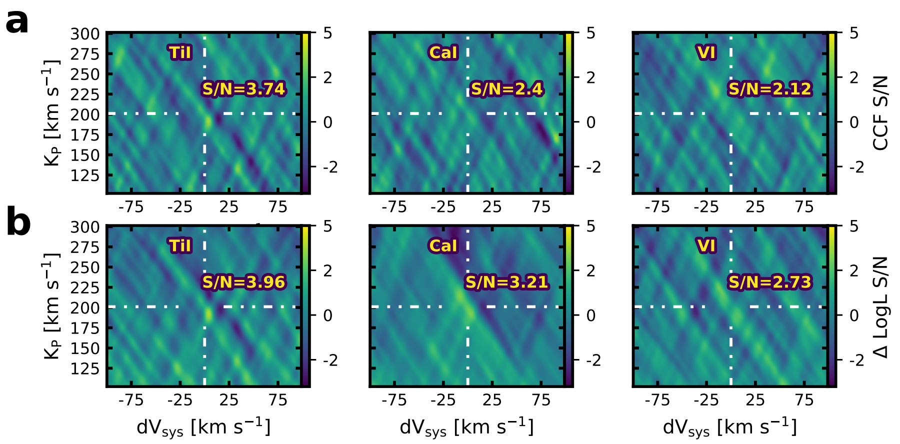

Supplementary Figure 2: **Summary of weak-to-non detections using the individual gas templates of Ti I,Ca I and V I.** **a** Cross correlation S/N maps for individual gas templates of Ti I,Ca I and V I. **b** These same S/N detection maps, but instead calculated using the log-likelihood formalism as described in ref [13].

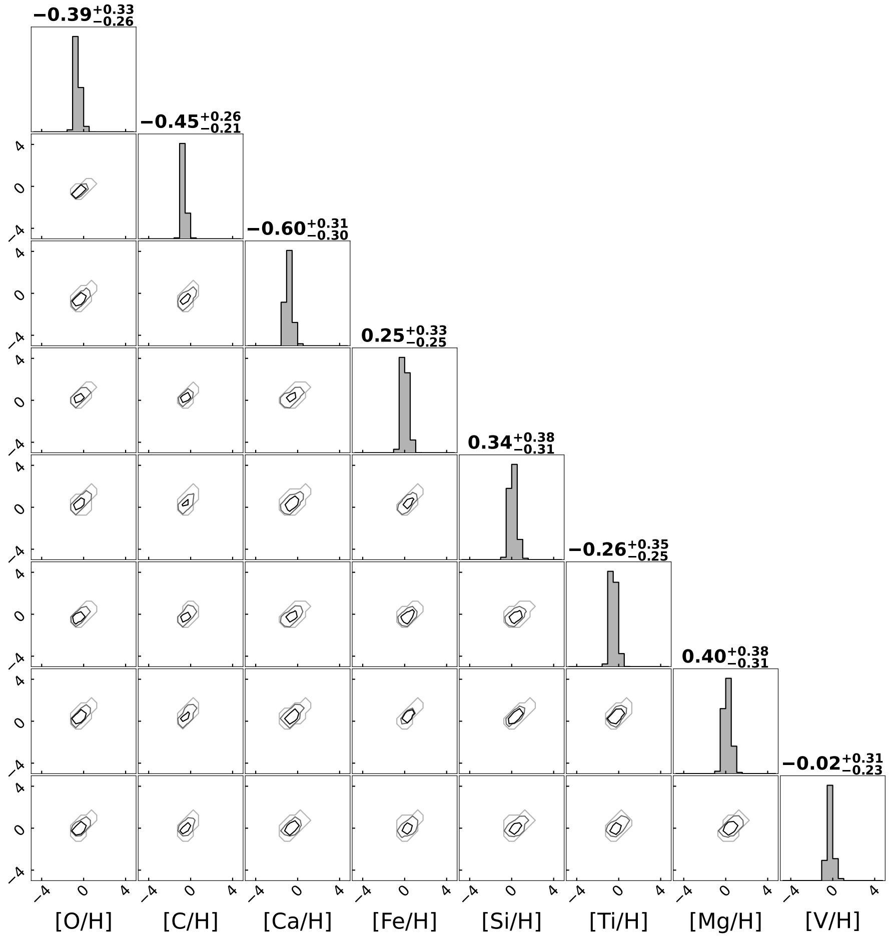

Supplementary Figure 3: **Summary of the elemental abundance ratio constraints from our main retrieval result**. The values here show the enrichment in the gas abundance, relative to solar, for each species in our model. The median and 68% confidence interval for each parameter is shown at the top of each panel. These values are also listed in column 3 of Supplementary Table 1.

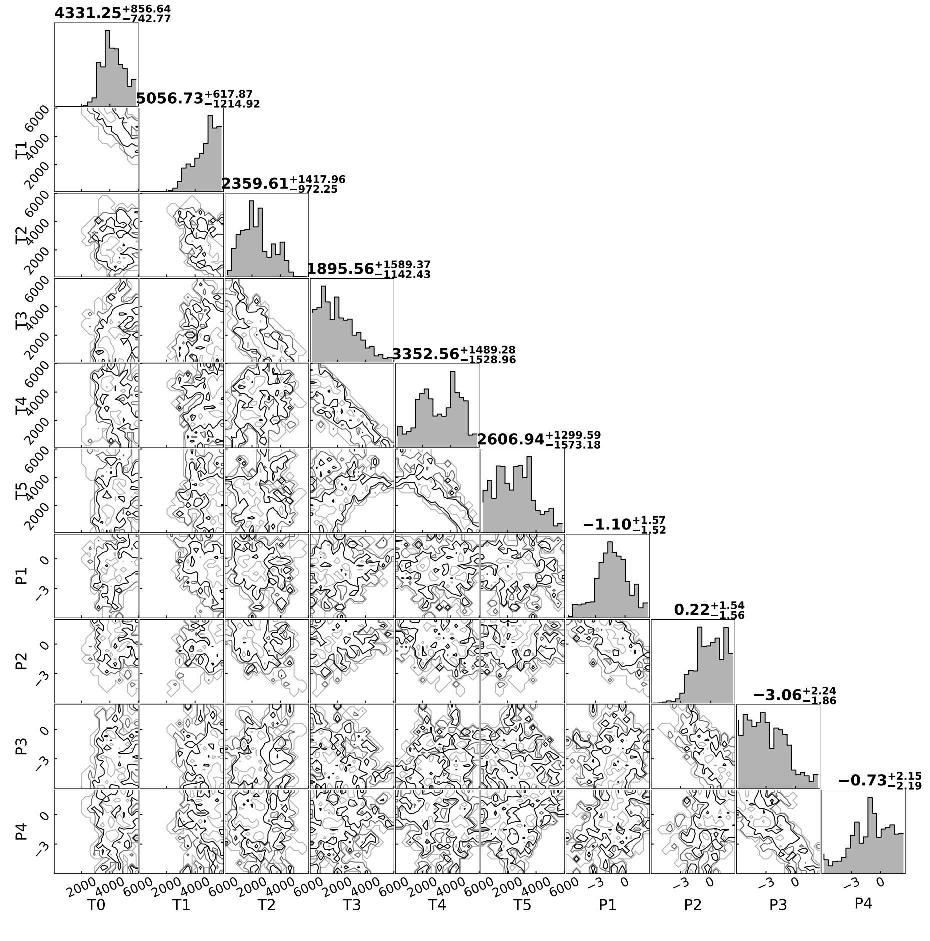

Supplementary Figure 4: **Summary of the PT-profile parameter constraints from our main retrieval result.** The median and 68% confidence interval for each parameter is shown at the top of each panel, in units of K.

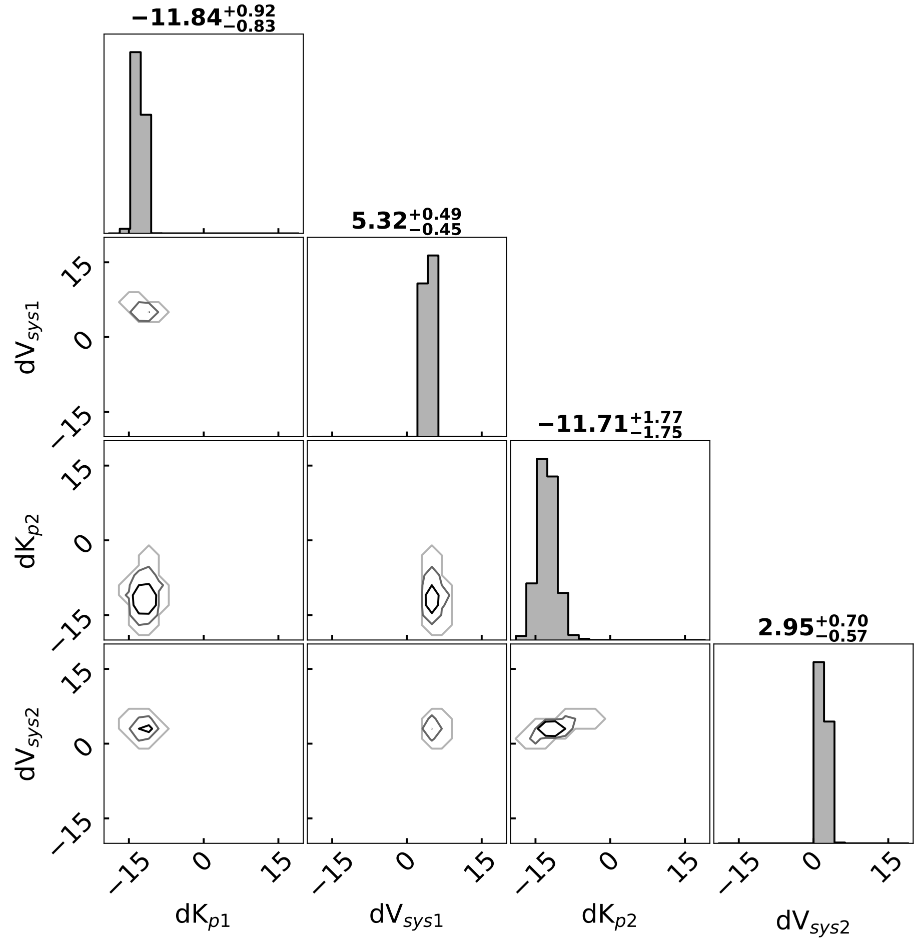

Supplementary Figure 5: **Summary of the velocity-offset parameters constraints from our main retrieval result.** The median and 68% confidence interval for each parameter is shown at the top of each panel, in units of km/s.

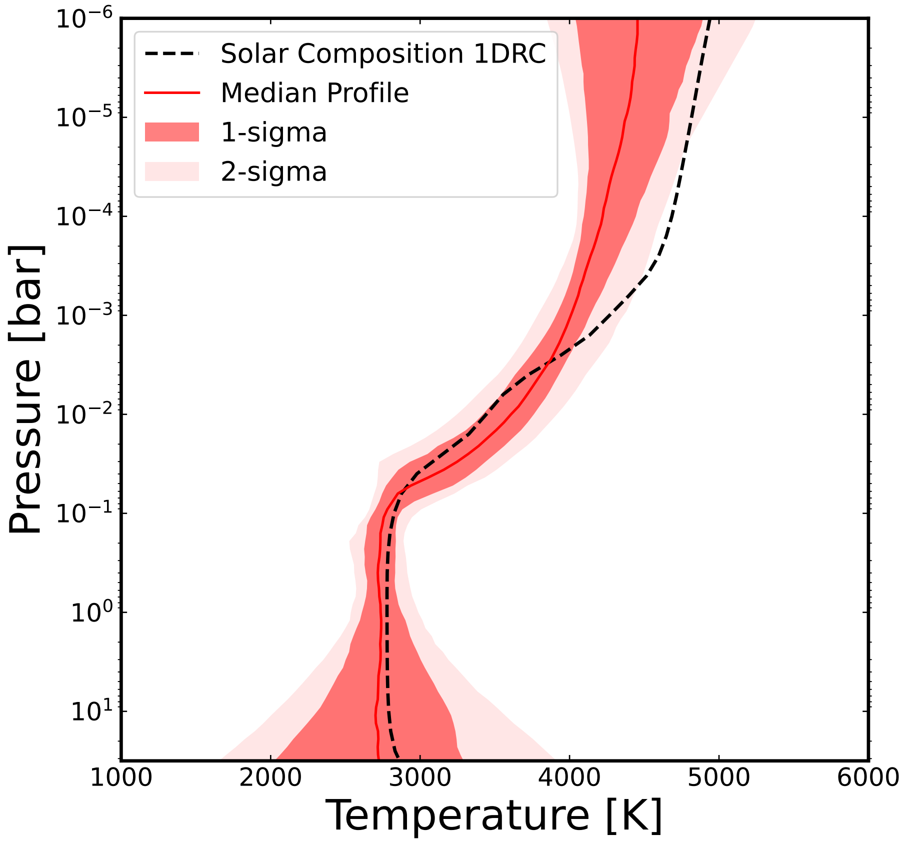

Supplementary Figure 6: **Pressure-Temperature profile for WASP-189b from our main retrieval result.** The median profile (red line) along with the 1 and 2σ confidence intervals around this median are shown, generated via 1000 random draws from the posterior distribution. We also show another P-T profile for WASP-189b generated under radiative-convective-thermochemical equilibrium conditions. Source data are provided as a Source Data file.

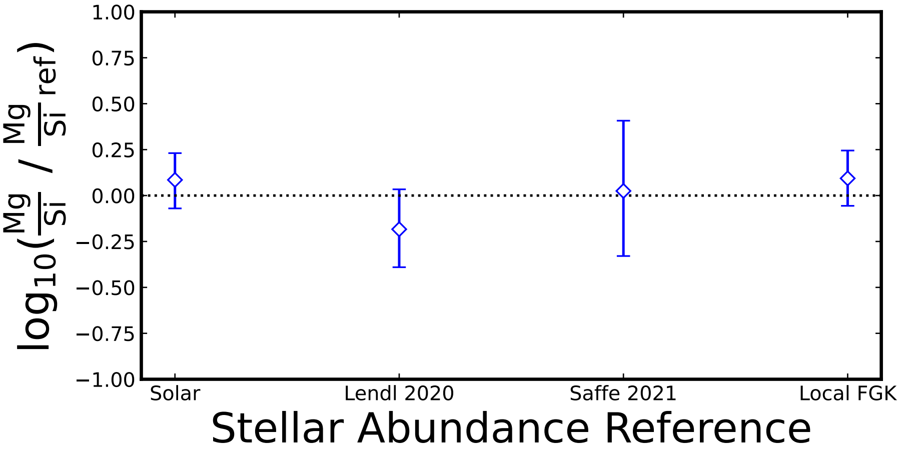

Supplementary Figure 7: **Logarithm of the Magnesium to Silicon (Mg/Si) ratio calculated in this analysis for the planet WASP-189b with respect to different astrophysical measurements of the ratio of Mg/Si**. The first tick shows the log10 ratio of Mg/Si in WASP-189b, measured in this work, over the Solar ratio of Mg/Si from ref [15]. The second and third ticks compute the log10 ratio of Mg/Si in the WASP-189b to two different references to the Mg/Si ratio of the host star WASP-189; tick mark Lendl 2020 is from ref [16], the main set of stellar abundances used in this analysis, and tick mark Saffe 2021 uses the computed Mg/Si ratio per the stellar abundances as reported in ref [17]. Finally, the tick Local FGK uses measured value in the local FGK population as cited in [33]. All errorbars represent the 68 % confidence interval on each measurement. Source data are provided as a Source Data file.

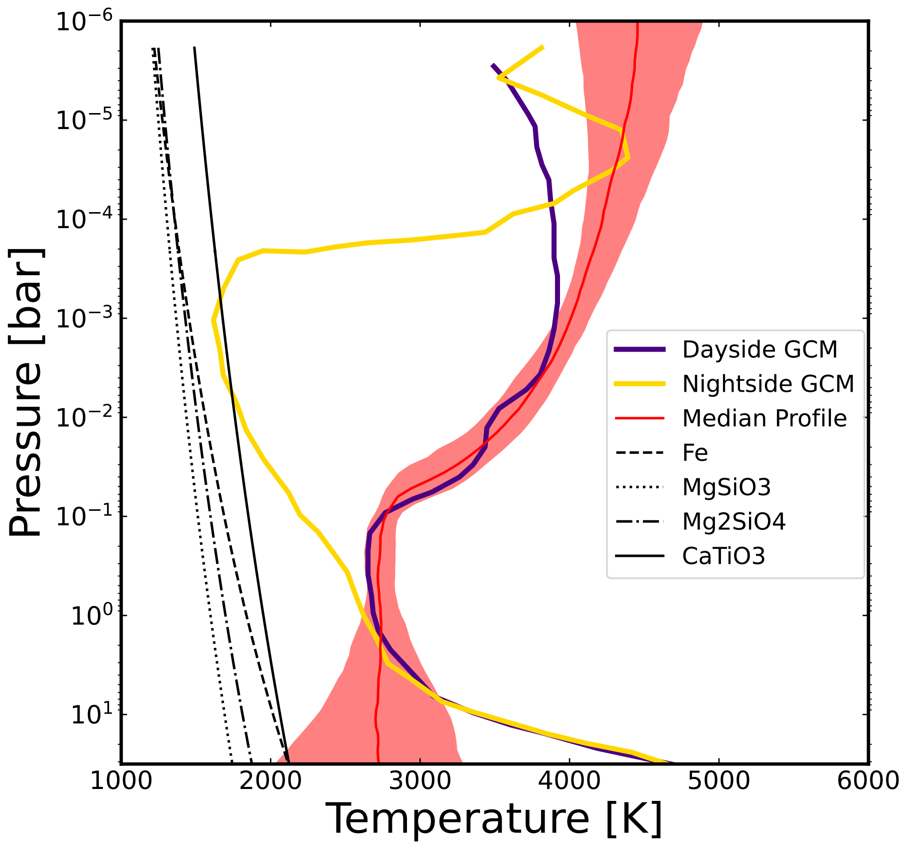

Supplementary Figure 8: **Thermal structure of WASP-189b with the 1σ confidence interval (red) as compared to condensation curves for Fe, Si, Ca and Ti bearing species (black lines)**. In yellow and indigo we show the vertical P-T profiles for the day-and-night sides for WASP-189b as predicted by the Global Circulation Models from [26]. Our retrieved thermal structure shows good agreement with the predictions for the day-side hemisphere of this ultra-hot Jupiter. Moreover, because our retrieved P-T profile does not cross the condensation curves of for these condensates, we do not expect the depletion of these species due to their condensation to bias our final results. Source data are provided as a Source Data file.

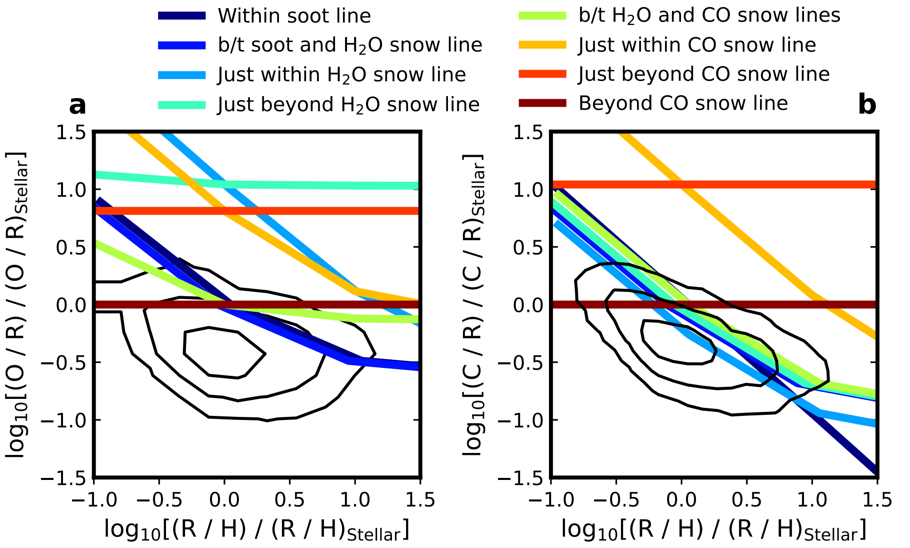

Supplementary Figure 9: **Predicted formation scenarios for WASP-189b based on our retrieved abundances.** **a** Oxygen to refractory abundance ratio in WASP-189b as a function of the total refractory content, relative to the stellar values calculated from [16]. **b** Same as **a** but with the carbon abundance instead of oxygen. The colored lines represent different formation predictions described in the modeling framework from [28]. The legend above indicates the predicted formation location within the disk that would produce the final observed combination of $[R/H]_*$ and $[O/R]_*$ or $[C/R]_*$. Our measured $[R/H]_*$, C/O and $[R/V]_*$ ratios are consistent with formation interior to the snow lines, with an accretion history of a stellar proportion of solids to gas, with those solids being slightly more rich in refractory to volatiles. However, the uncertainties on our $[R/V]_*$ measurement means we cannot rule out formation beyond the CO snow line. This ambiguity highlights the difficulty in predicting exact formation locations within the protoplanetary disk for hot Jupiters [14, 34]. The 2D histogram show the 39.3, 86.4 and 98.9% joint probability contours for these quantities. Source data are provided as a Source Data file.

Supplementary Tables

Element	log(X/H)	$[X/H]_{\odot}$	$[X/H]_{*L20}$	$[X/H]_{*S21}$	$[X/H]_{*La24}$
O	$-3.69^{+0.33}_{-0.26}$	$-0.39^{+0.33}_{-0.26}$	$-0.38^{+0.36}_{-0.27}$	$-$	-
C	$-3.99^{+0.26}_{-0.21}$	$-0.45^{+0.26}_{-0.21}$	$-0.31^{+0.29}_{-0.23}$	$-0.02^{+0.33}_{-0.27}$	-
Ca	$-6.30^{+0.31}_{-0.30}$	$-0.60^{+0.31}_{-0.30}$	$-0.85^{+0.29}_{-0.23}$	$-0.67^{+0.46}_{-0.45}$	$-1.08^{+0.32}_{-0.32}$
Fe	$-4.28^{+0.33}_{-0.25}$	$0.25^{+0.33}_{-0.25}$	$-0.08^{+0.35}_{-0.29}$	$0.22^{+0.36}_{-0.30}$	$-0.29^{+0.35}_{-0.28}$
Si	$-4.14^{+0.38}_{-0.31}$	$0.34^{+0.38}_{-0.31}$	$0.20^{+0.38}_{-0.31}$	$0.27^{+0.41}_{-0.38}$	-
Ti	$-7.29^{+0.35}_{-0.25}$	$-0.26^{+0.35}_{-0.25}$	$-0.02^{+0.38}_{-0.25}$	$-0.29^{+0.36}_{-0.28}$	$-0.56^{+0.35}_{-0.27}$
Mg	$-4.05^{+0.37}_{-0.30}$	$0.40^{+0.38}_{-0.31}$	$-0.01^{+0.39}_{-0.33}$	$0.29^{+0.44}_{-0.39}$	$-0.30^{+0.31}_{-0.30}$
V	$-8.12^{+0.31}_{-0.23}$	$-0.02^{+0.31}_{-0.23}$	-	-	-

Supplementary Table 1: **Retrieved abundance pattern measured in the atmosphere of WASP-189b.** The first column shows each elemental species in our model. Column 2 has the abundance ratio of log(X/H) for each element, X, measured in the atmosphere of WASP-189b. Columns 3, 4, 5 and 6 show these ratios as compared to both solar and various literature stellar values. Column 3 is the log(X/H) relative to the solar value [15] (the main output of the retrieval). (To calculate column 2 from column 3, we add each value of column 3 with the $\log10(\frac{X}{H})$ solar value as reported in [15]). Column 4 are these ratios relative to the stellar values of ref [16] (L20). Column 5 are ratios relative to the values reported in ref [17] (S21) and column 6 the values from ref [18] (La24). When the abundance of a species is not reported, it is not listed. For all stellar species with reported uncertainties, the shown uncertainties reflect the 68% confidence interval.

References

[1] Karman, T. *et al.* Update of the HITRAN collision-induced absorption section. *Icarus* **328**, 160–175 (2019).

[2] Polyansky, O. L. *et al.* ExoMol molecular line lists XXX: a complete high-accuracy line list for water. *MNRAS* **480**, 2597–2608 (2018). 1807.04529.

[3] Li, G. *et al.* Rovibrational Line Lists for Nine Isotopologues of the CO Molecule in the X $^1\Sigma^+$ Ground Electronic State. *ApJS* **216**, 15 (2015).

[4] Rothman, L. S. *et al.* HITEMP, the high-temperature molecular spectroscopic database. *J. Quant. Spec. Radiat. Transf.* **111**, 2139–2150 (2010).

[5] Kurucz, R. L. Including All the Lines: Data Releases for Spectra and Opacities through 2017. In *Workshop on Astrophysical Opacities*, vol. 515 of *Astronomical Society of the Pacific Conference Series*, 47 (2018).

[6] John, T. L. Continuous absorption by the negative hydrogen ion reconsidered. *A&A* **193**, 189–192 (1988).

[7] Gandhi, S. *et al.* Retrieval Survey of Metals in Six Ultrahot Jupiters: Trends in Chemistry, Rain-out, Ionization, and Atmospheric Dynamics. *AJ* **165**, 242 (2023). 2305.17228.

[8] Prinoth, B. *et al.* Titanium oxide and chemical inhomogeneity in the atmosphere of the exoplanet WASP-189 b. *Nature Astronomy* **6**, 449–457 (2022). 2111.12732.

[9] Lesjak, F. *et al.* Retrieving wind properties from the ultra-hot dayside of WASP-189b with CRIRES$^+$. *arXiv e-prints* arXiv:2411.19662 (2024). 2411.19662.

[10] Arcangeli, J. *et al.* H^- Opacity and Water Dissociation in the Dayside Atmosphere of the Very Hot Gas Giant WASP-18b. *ApJL* **855**, L30 (2018). 1801.02489.

[11] Bell, T. J. & Cowan, N. B. Increased Heat Transport in Ultra-hot Jupiter Atmospheres through H_2 Dissociation and Recombination. *ApJL* **857**, L20 (2018). 1802.07725.

[12] Mansfield, M. *et al.* A unique hot Jupiter spectral sequence with evidence for compositional diversity. *Nature Astronomy* **5**, 1224–1232 (2021). 2110.11272.

[13] Line, M. R. *et al.* A solar c/o and sub-solar metallicity in a hot jupiter atmosphere. *Nature* **598**, 580–584 (2021).

[14] Smith, P. C. B. *et al.* The Roasting Marshmallows Program with IGRINS on Gemini South. II. WASP-121 b has Superstellar C/O and Refractory-to-volatile Ratios. *AJ* **168**, 293 (2024). 2410.19017.

[15] Asplund, M., Amarsi, A. M. & Grevesse, N. The chemical make-up of the Sun: A 2020 vision. *A&A* **653**, A141 (2021). 2105.01661.

[16] Lendl, M. *et al.* The hot dayside and asymmetric transit of WASP-189 b seen by CHEOPS. *A&A* **643**, A94 (2020). 2009.13403.

[17] Saffe, C. *et al.* Chemical analysis of early-type stars with planets. *A&A* **647**, A49 (2021). 2101.04416.

[18] Lam, M. B., Hoeijmakers, H. J., Prinoth, B. & Thorsbro, B. Secrets in the shadow: High precision stellar abundances of fast-rotating A-type exoplanet host stars through transit spectroscopy. *A&A* **691**, A141 (2024). 2409.15951.

[19] Asplund, M., Grevesse, N., Sauval, A. J. & Scott, P. The Chemical Composition of the Sun. *ARA&A* **47**, 481–522 (2009). 0909.0948.

[20] Fortney, J. J., Lodders, K., Marley, M. S. & Freedman, R. S. A Unified Theory for the Atmospheres of the Hot and Very Hot Jupiters: Two Classes of Irradiated Atmospheres. *ApJ* **678**, 1419–1435 (2008). 0710.2558.

[21] Ramkumar, S., Gibson, N. P., Nugroho, S. K., Maguire, C. & Fortune, M. High-resolution emission spectroscopy retrievals of MASCARA-1b with CRIRES+: strong detections of CO, H_2O, and Fe emission lines and a C/O consistent with solar. *MNRAS* **525**, 2985–3005 (2023). 2308.07157.

[22] Brogi, M. *et al.* The Roasting Marshmallows Program with IGRINS on Gemini South I: Composition and Climate of the Ultrahot Jupiter WASP-18 b. *AJ* **165**, 91 (2023). 2209.15548.

[23] Yan, F. *et al.* A temperature inversion with atomic iron in the ultra-hot dayside atmosphere of WASP-189b. *A&A* **640**, L5 (2020). 2007.02716.

[24] Wakeford, H. R. *et al.* HAT-P-26b: A Neptune-mass exoplanet with a well-constrained heavy element abundance. *Science* **356**, 628–631 (2017). 1705.04354.

[25] Visscher, C., Lodders, K. & Fegley, B., Jr. Atmospheric Chemistry in Giant Planets, Brown Dwarfs, and Low-mass Dwarf Stars. III. Iron, Magnesium, and Silicon. *ApJ* **716**, 1060–1075 (2010). 1001.3639.

[26] Lee, E. K. H. *et al.* The Mantis Network II: examining the 3D high-resolution observable properties of the UHJs WASP-121b and WASP-189b through GCM modelling. *MNRAS* **517**, 240–256 (2022). 2210.11986.

[27] Prinoth, B. *et al.* Time-resolved transmission spectroscopy of the ultra-hot Jupiter WASP-189 b. *A&A* **678**, A182 (2023). 2308.04523.

[28] Chachan, Y., Knutson, H. A., Lothringer, J. & Blake, G. A. Breaking Degeneracies in Formation Histories by Measuring Refractory Content in Gas Giants. *ApJ* **943**, 112 (2023). 2211.09080.

[29] Wang, Y., lin Zhou, J., hui gen, L. & Meng, Z. Forming different planetary architectures. i. the formation efficiency of hot jupiters from high-eccentricity mechanisms. *The Astrophysical Journal* **848**, 20 (2017). URL https://dx.doi.org/10.3847/1538-4357/aa8868.

[30] Albrecht, S. H., Dawson, R. I. & Winn, J. N. Stellar Obliquities in Exoplanetary Systems. *PASP* **134**, 082001 (2022). 2203.05460.

[31] Rice, M., Wang, S. & Laughlin, G. Origins of Hot Jupiters from the Stellar Obliquity Distribution. *ApJL* **926**, L17 (2022). 2201.11768.

[32] Fortney, J. J., Dawson, R. I. & Komacek, T. D. Hot Jupiters: Origins, Structure, Atmospheres. *Journal of Geophysical Research (Planets)* **126**, e06629 (2021). 2102.05064.

[33] Brewer, J. M. & Fischer, D. A. C/O and Mg/Si Ratios of Stars in the Solar Neighborhood. *ApJ* **831**, 20 (2016).

[34] Lothringer, J. D. *et al.* Refractory and Volatile Species in the UV-to-IR Transmission Spectrum of Ultra-hot Jupiter WASP-178b with HST and JWST. *AJ* **169**, 274 (2025). 2503.15472.
